# CT Radiomics Model for Discriminating the Risk Stratification of Gastrointestinal Stromal Tumors: A Multi-Class Classification and Multi-Center Study

**DOI:** 10.3389/fonc.2021.654114

**Published:** 2021-06-08

**Authors:** Zhonghua Chen, Linyi Xu, Chuanmin Zhang, Chencui Huang, Minhong Wang, Zhan Feng, Yue Xiong

**Affiliations:** ^1^ Department of Radiology, Haining People’s Hospital, Jiaxing, China; ^2^ Department of Research Collaboration, R&D Center, Beijing Deepwise & League of PHD Technology Co., Ltd, R&D Center, Beijing, China; ^3^ Department of Radiology, First Affiliated Hospital of Wannan Medical College, Wuhu, China; ^4^ Department of Radiology, First Affiliated Hospital, School of Medicine, Zhejiang University, Hangzhou, China

**Keywords:** gastrointestinal stromal tumor, risk classification, radiomics, computed tomography, multi-class classification

## Abstract

**Objective:**

To establish and verify a computed tomography (CT)-based multi-class prediction model for discriminating the risk stratification of gastrointestinal stromal tumors (GISTs).

**Materials and Methods:**

A total of 381 patients with GISTs were confirmed by surgery and pathology. Information on 213 patients were obtained from one hospital and used as training cohort, whereas the details of 168 patients were collected from two other hospitals and used as independent validation cohort. Regions of interest on CT images of arterial and venous phases were drawn, radiomics features were extracted, and dimensionality reduction processing was performed. Using a one-vs-rest method, a Random Forest-based GISTs risk three-class prediction model was established, and the receiver operating characteristic curve (ROC) was used to evaluate the performance of the multi-class classification model, and the generalization ability was verified using external data.

**Results:**

The training cohort included 96 very low-risk and low-risk, 60 intermediate-risk and 57 high-risk patients. External validation cohort included 82 very low-risk and low-risk, 48 intermediate-risk and 38 high-risk patients. The GISTs risk three-class radiomics model had a macro/micro average area under the curve (AUC) of 0.84 and an accuracy of 0.78 in the training cohort. It had a stable performance in the external validation cohort, with a macro/micro average AUC of 0.83 and an accuracy of 0.80.

**Conclusion:**

CT radiomics can discriminate GISTs risk stratification. The performance of the three-class radiomics prediction model is good, and its generalization ability has also been verified in the external validation cohort, indicating its potential to assist stratified and accurate treatment of GISTs in the clinic.

## Introduction

Gastrointestinal stromal tumors (GISTs) originate from the interstitial cells of the gastrointestinal pacemaker Cajal cells, which are the most common mesenchymal tissue-derived tumors in the digestive system. These usually occur in the stomach and small intestine, accounting for approximately 1 to 2% of all malignant tumors of the digestive tract (1). GISTs have diverse biological behaviors and generally considered to be potentially malignant (2, 3).

Many studies have confirmed that tumor site and size, mitotic count, and tumor rupture are independent prognostic factors for GISTs (4). Joensuu et al. proposed a modified version of the National Institutes of Health (NIH) risk stratification standard, which integrates these four prognostic factors into an evaluation system and classifies the risk of GISTs into four levels: very low, low, intermediate, and high. It is currently a clinical stratification standard for predicting the risk of recurrence with relatively high practicality (5).

Due to the lack of specific clinical manifestations of GISTs, preoperative diagnosis and identification mainly rely on computed tomography (CT) examinations. Radiomics can extract quantitative features in images with high throughput and convert these into mineable data, which continuously play a role in the entire process of tumor detection, diagnosis, prognosis, and follow-up (6, 7). Several studies on radiomics in the risk stratification of GISTs have been conducted, but most of the previous studies were based on single-center data, and few investigations have verified the radiomics model using independent external data (8–10). Radiomics parameters are influenced by scanning equipment and scanning parameters to varying degrees; single-center studies have serious limitations, and prediction models may have varying degrees of overfitting (11). In addition, generally, previous studies only distinguished low-malignant (very low, low) and high-malignant (intermediate, high) GISTs (12–14). In fact, the recurrence risk of high-risk GISTs is significantly higher than intermediate-risk GISTs, and targeted therapies of the two are not the same (15). More refined predictions can better fit clinical needs.

Therefore, to solve the above mentioned problems, this study collected multi-center image data and constructed a GISTs risk stratification three-class preoperative prediction model based on CT radiomics and evaluated the generalization ability of the prediction model using independent external validation data sets to provide accurate auxiliary tools for the stratified treatment of GISTs in the clinic.

## Materials and Methods

### Characteristics of Patients

This study collected 381 patients with GISTs from January 1, 2016 to July 1, 2020 from three hospitals. Among these, 213 data from The First Affiliated Hospital of Wannan Medical College were used as the training group, and 168 data from two other hospitals were used for external validation. The inclusion criteria were as follows: (1) patients were pathologically diagnosed as GISTs; (2) the patient’s enhanced CT examination was within 15 days before surgery; (3) the patient’s pathological results had clear risk stratification. The exclusion criteria were as follows: (1) the patients received neoadjuvant treatment with imatinib or other tyrosine kinase inhibitors before surgery; and (2) no preoperative contrast-enhanced CT or poor CT image quality (e.g. presence of artifacts).

The clinical characteristics of the GISTs patients included sex, age, and tumor site. GISTs risk stratification adopted the modified version of the NIH risk stratification standard. Patients were divided into three groups: low-risk and very low-risk, intermediate-risk, and high-risk. This study was a retrospective study, and the patient’s informed consent was thereby waived, as approved by the hospital ethics committee.

### CT Image Acquisition

The Brilliance 64 spiral CT and Brilliance 256 spiral CT from Philips Electronics, Ltd., The Netherlands, and Siemens SOMATOM Definition dual-source CT were used. All patients received conventional abdominal CT scan. Detailed information of the CT protocol is shown in **Table 1**. The three-phase CT scan included conventional plain scan and 25–30 s arterial phase scan and 55–60 s venous phase scan after contrast injection. Using a high-pressure syringe from Ulm, Germany (Ulrich CT Plus 150, Ulrich Medical, Ulm, Germany), 70–100 ml of contrast agent (Ioversol 350, Heng Rui Pharma, Jiangsu, China) was injected through the anterior elbow vein at a rate of 2.5–3.5 ml/s.

**Table 1:** The protocols of the CT scan for the patients with GISTs.

Manufacture	Philips	SIEMENS	Philips
CT scanner	Brilliance 64	Dual source CT	Brilliance 256
Tube voltage (kV)	120	120	120
Tube current (mA)	250	200	250
Rotation time (s)	0.4	0.5	0.5
Detector collimation (mm)	64 × 0.625	128 × 0.6	64 × 0.625
Pitch	0.891	0.6	0.914
Slice thickness (mm)	5	5	5
Slice spacing (mm)	5	5	5
Matrix	512 × 512	512 × 512	512 × 512
Field of view (mm)	350	300	350
Algorithm	standard	standard	standard

CT, Computed tomography; GISTs, Gastrointestinal stromal tumors.

### Tumor Segmentation

**Figure 1:**
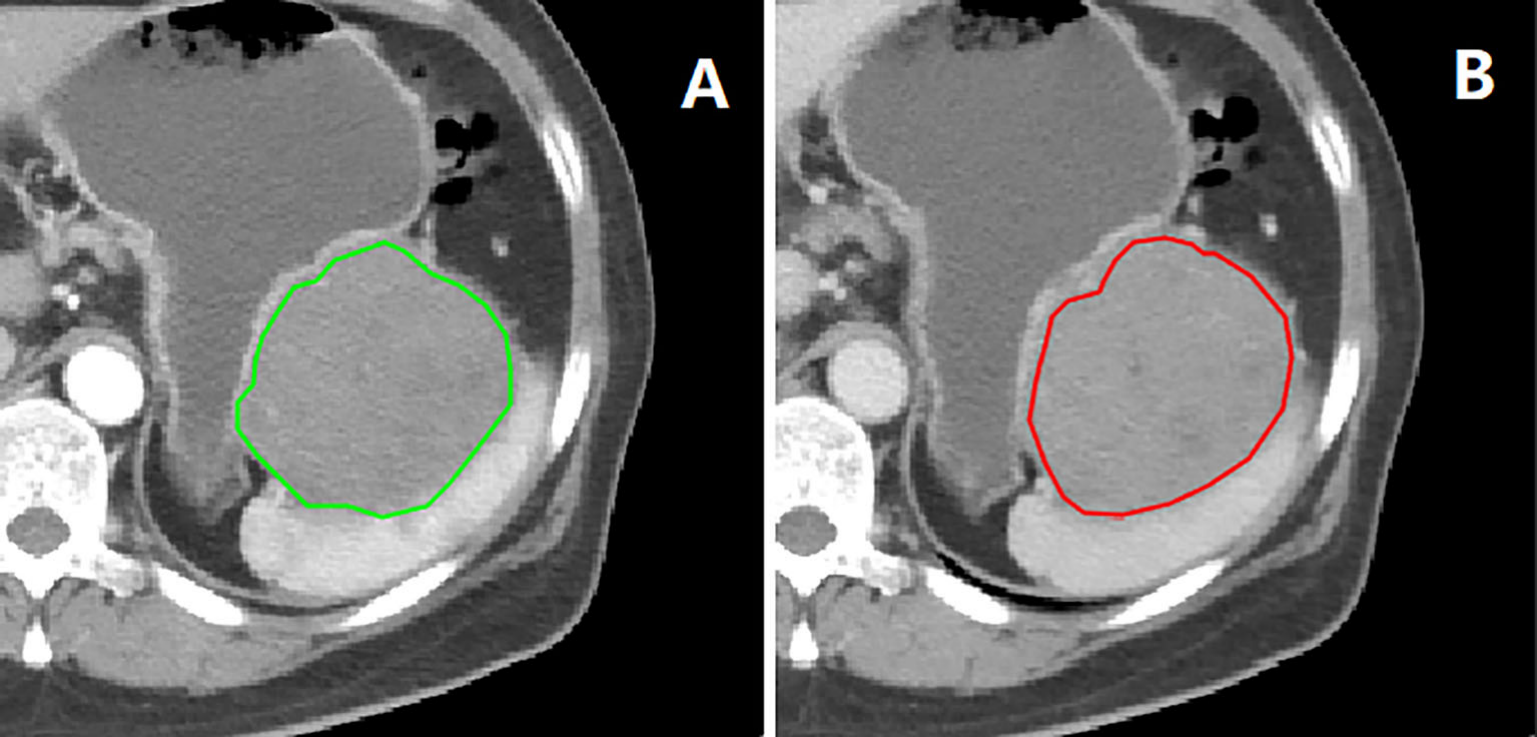
Example of tumor delineation and segmentation. **(A)** arterial phase; **(B)** venous phase.

Tumor segmentation and radiomics feature extraction were both based on MATLAB’s IBEX software package (16). The regions of interest (ROI) segmentation was performed by one radiologist and confirmed by another. The ROIs were delineated slice by slice along the inner edge of the tumor contour on the CT images of the arterial and venous phases before GISTs surgery (**Figure 1**). Both radiologists were blinded to GIST risk stratification before ROI segmentation. Because the boundary of the lesion cannot be accurately identified from the plain scan image, it was not used in this study.

Preprocessing was performed before feature extraction, including image resampling and gray value normalization. Radiomics parameters include six categories: histogram parameters, 2.5D and 3D gray level co-occurrence matrix, neighborhood gray-tone difference matrix, gray level run length matrix, and shape. There were 704 radiomics parameters extracted from the lesions at each stage and a total of 1,408 radiomics parameters from two stages of each patient.

### Radiomics Feature Selection and Model Building

In this study, Spearman correlation analysis was performed on the multicollinearity of the features, and the correlation coefficient threshold was 0.8. Then, based on the impurity-based feature importance in the tree model, feature dimensionality reduction was performed. After that, a Random Forest classifier was used to establish the prediction model, and a 10-fold cross-validation on the model was conducted in the training cohort. Finally, the generalization ability was evaluated in the independent external validation cohort.

Research on multi-class classification radiomics is relatively rare. The basic idea of multi-class classification problems in machine learning is disassembly, which means that multi-class classification tasks are split into several binary classification tasks to solve. This study adopted a one-vs-rest method that is commonly used in multi-class classification. For example, when the high-risk group is marked as positive, the remaining two groups of data are regarded as negative. Similarly, the intermediate-risk group, very low-risk and low-risk group are also used as the positive classes, and a total of three one-vs-rest classifiers are trained. The performance of the model was evaluated on the basis of receiver operating characteristic (ROC) curves and the area under the curve (AUC), and finally, the generalization ability of each model was evaluated in the external validation cohort. In addition to assessing the degree of discrimination of one-vs-rest for the risk of each group, the multi-class classification model also needs to be evaluated as a whole. Due to different calculation methods of multiple confusion matrix aggregation, the performance indicators of the multi-class classification were divided into macro-average and micro-average, so the global performance indicators of the multi-class classification in this study included sensitivity (macro average/micro average), specificity (macro average/micro average), F1 score (macro average/micro average), and AUC (macro average/micro average).

### Statistical Analysis

All statistical analyses were performed with R software (version 3.4.1; http://www.Rproject.org) and Python (version 3.8.5; https://www.python.org). Quantitative data was described by mean ± standard deviation, and qualitative data was described by frequency (percent). Qualitative variables were compared using chi-square test. Continuous variable data was evaluated using a two-sample t-test or Wilcoxon test. *p* <0.05 was considered statistically significant.

## Results

A total of 381 patients with GISTs were enrolled in this study, including a training cohort of 119 men and 94 women, and an external validation cohort of 89 men and 79 women. **Table 2** shows the clinical data of training cohort and external validation cohort. There were no significant statistical differences between training cohort and external validation cohort in terms of age, sex, risk stratification and site of GISTs.

**Table 2:** Patient characteristics in the training and external validation cohorts.

	Training cohort	External validation cohort	*p* value
Age (years)	57.81 ± 10.13	55.86 ± 10.74	0.68
Sex (n, %)			0.65
Male	119 (55.9)	89 (53.0)	
Female	94 (44.1)	79 (47.0)	
Tumor size (cm)	5.45 ± 1.67	4.87 ± 1.62	0.72
Risk classification (n, %)			0.63
Very low and low risk	96 (45.1)	82 (48.8)	
Intermediate risk	60 (28.2)	48 (28.6)	
High risk	57 (26.7)	38 (22.6)	
Site (n, %)			0.48
Gastric	85 (39.9)	74 (44.0)	
Intestinal	128 (60.1)	94 (66.0)	

p < 0.05 indicates that difference is statistically significant.

**Figure 2:**
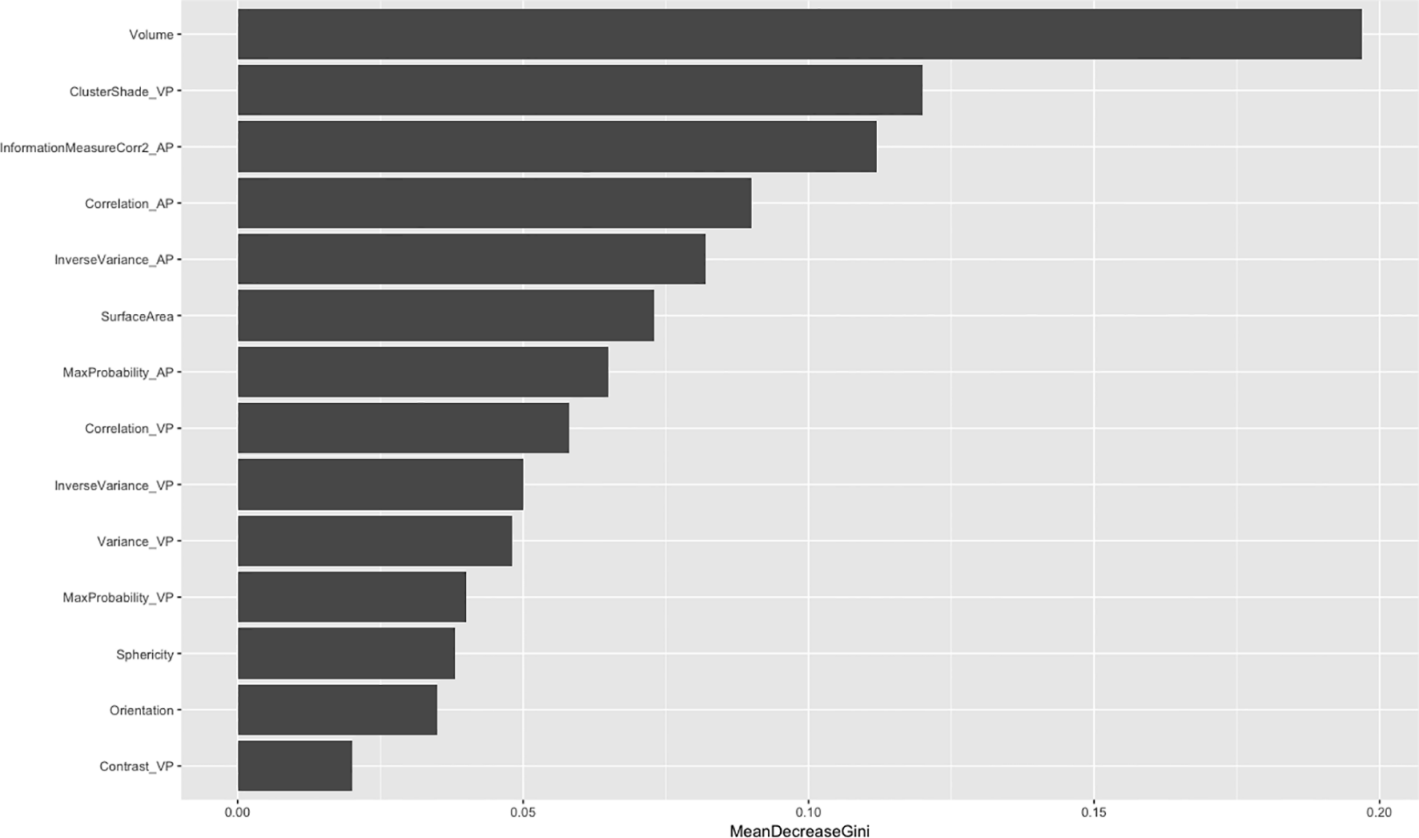
The importance of radiomics features. AP, arterial phase; VP, venous phase.

After feature dimensionality reduction, the final 14 features were used in modeling. Among these, nine gray-level co-occurrence matrices, four morphology features, and only one neighborhood gray-tone difference matrix were selected (**Figure 2**). Among the 10 texture features, six parameters were from venous phase and four from artistic phase.

**Figure 3:**
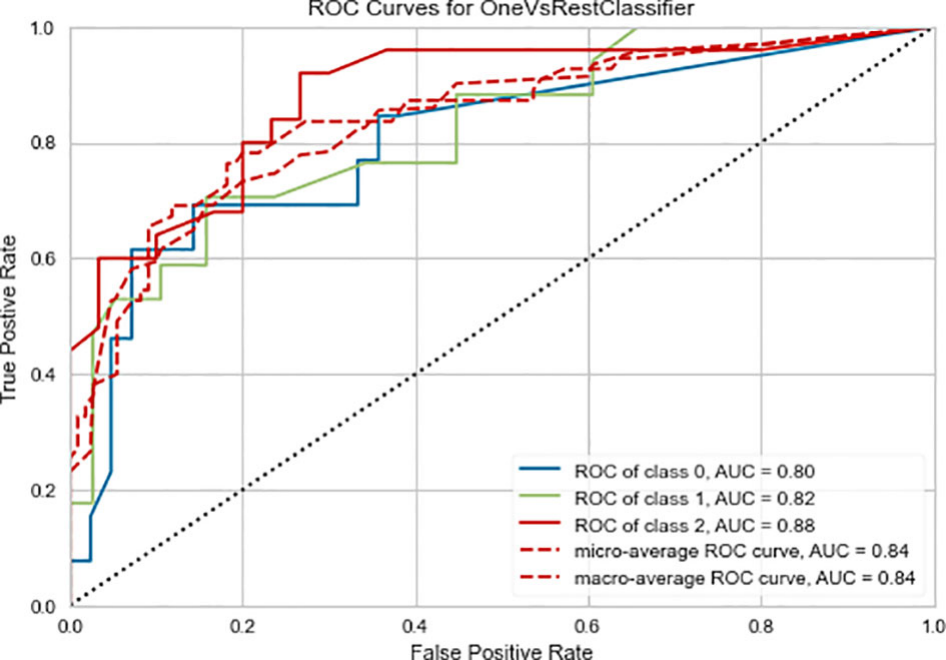
Three-class (one-*vs*-rest) Receiver operating characteristic (ROC) curve of the training cohort of the radiomics prediction model. Class 0 is the very low- and low-risk group, class 1 is the intermediate-risk group, and class 2 is the high-risk group. The two dashed lines respectively show the ROC curves of micro-average and macro-average, indicating the overall distinguishing ability of the three-class classification.

**Figure 4:**
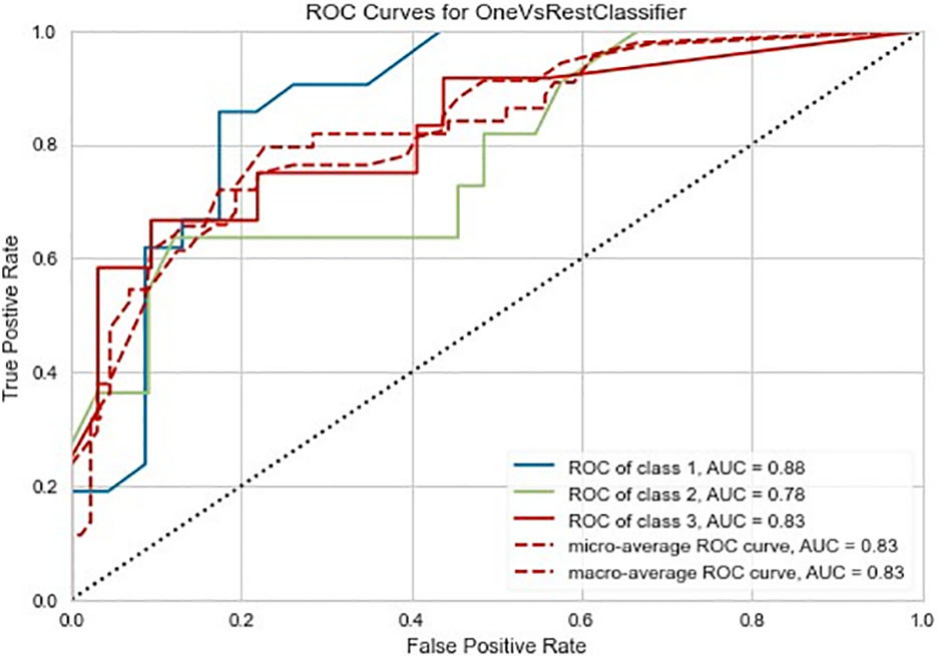
Three-class (one-vs-rest) Receiver operating characteristic (ROC) diagram of the external validation cohort of the radiomics prediction model. Class 0 is the very low- and low-risk group, class 1 is the intermediate-risk group, and class 2 is the high-risk group. The two dashed lines respectively show the ROC curves of micro-average and macro-average, indicating the overall distinguishing ability of the three-class classification.

The Random Forest-based three-class prediction model showed good overall performance using the training cohort, with a macro/micro average AUC of 0.84 and an accuracy of 0.78. The degree of discrimination AUC was 0.80 for the very low- and low-risk group, 0.82 for the intermediate-risk group, and 0.88 for the high-risk group. The model had a stable performance in the external validation cohort, with a macro/micro average AUC of 0.83, and an accuracy of 0.80. Among these, the AUC of the very low- and low-risk group was 0.88, 0.78 for the intermediate-risk group, and 0.83 for the high-risk group (**Table 3** and **Figures 3**, **4**).

**Table 3:** The predictive performance of radiomics model for discrimination of the three different risk degrees of GISTs.

	Overall performance (macro/micro)		Very low and low risk		Intermediate risk		High risk	
	Training cohort	External Validated cohort	Training cohort	External Validated cohort	Training cohort	External Validated cohort	Training cohort	External Validated cohort
Accuracy	0.78	0.80	0.80	0.83	0.74	0.75	0.82	0.82
Sensitivity	0.61/0.65	0.65/0.70	0.62	0.88	0.67	0.60	0.80	0.55
Specificity	0.79/0.83	0.84/0.85	0.86	0.79	0.82	0.80	0.77	0.94
F1 score	0.64/0.69	0.66/0.70	0.80	0.83	0.55	0.56	0.61	0.62
AUC	0.84/0.84	0.83/0.83	0.80	0.88	0.82	0.78	0.88	0.83

GISTs, Gastrointestinal stromal tumors; AUC, area under the receiver operating characteristic.

## Discussion

In this study, we established a three-class radiomics prediction model for the risk stratification of GISTs and found that the prediction model based on Random Forest not only showed excellent ability to distinguish the risk stratification of GISTs in the training cohort, but was also highly capable of conducting generalization of external independent validation data.

GISTs have complex and unpredictable biological behaviors. *KIT* or platelet-derived growth factor receptor α (*PDGFRA*) functional gene mutations and activation of downstream signaling pathways are the main pathogenetic pathways of GISTs (17). Imatinib, a small-molecule inhibitor of tyrosine kinase, can be combined with *KIT* and *PDGFRA*, and is currently the first-line molecular targeted drug for the treatment of GISTs (18). The current guidelines recommend that the adjuvant treatment of high-risk GISTs patients should be more than three years, but for the treatment of imatinib in intermediate-risk GISTs patients, there is no unified guideline, and the duration of adjuvant therapy is unclear (19). Patients with low malignant potential (very low and low risk) generally have good tumor prognosis, most of who can be cured only by surgery and do not require further adjuvant imatinib therapy (20–22). According to the current diagnosis and treatment paradigm, individualized preoperative prediction of the recurrence risk of GISTs is particularly important. Based on the risk stratification, the risk of GISTs patients can be stratified to achieve individualized treatment of patients.

In recent years, with the development of radiomics, radiology research on GISTs risk stratification has also followed an objective and quantitative direction. Currently, most GISTs radiomics research focuses on risk stratification prediction, and results suggest that the radiomics method is better than traditional visual analysis (9, 10, 13). Currently, most GISTs risk stratification radiomics studies do not distinguish intermediate-risk and high-risk GISTs, but the recurrence risk and adjuvant treatment plan of high-risk GISTs are obviously different from those of intermediate-risk patients, and high-risk GISTs have become highly relevant. Zhang et al. conducted a four-class prediction study on the risk of GISTs, and the results suggested that the training cohort AUC was 0.86 with an accuracy of 0.65, and the internal validation cohort AUC was 0.80, with an accuracy of 0.67, proving a good prediction performance of the model (13). However, there were only eight very low risk cases in this study, and the sample size among the four categories was unbalanced. In this study, the number of very low-risk cases in the training cohort was only 17, which is relatively small compared to the other groups. Because very low-risk patients have a relatively good prognosis, and the clinical significance of distinguishing between very low risk and low risk is not significant, we combined the very low-risk and low-risk into one group, and only conducted a three-class prediction study. In addition, Zhang et al.’s four-class study used a one-vs-one multi-class approach. Their ROC chart in the results showed that the degree of discrimination between intermediate-risk and high-risk GISTs was not ideal. Our results showed that the one-vs-rest ROC curves of the three GISTs groups all had good distinguishing ability, and the unique global performance indicators of the multi-class classification also reflected the comprehensive and excellent predictive performance of this model, which again confirmed the links between radiomics and GISTs pathological manifestations. Radiomics can be well matched with the pathological stratification of GISTs risk, realizing risk multi-class image prediction before surgery and fitting the practical clinical needs.

Among the many GISTs image studies, only a few have verified the prediction models with external data (8). Most studies do not have multi-center data, but simply divide the single-center data into a training cohort and a validation cohort for internal verification. Studies have confirmed that there are huge differences in image scanning, post-processing reconstruction algorithms, and scanning parameter settings in equipment from different manufacturers. These factors influence the image and finally cause significant differences in radiomics parameters (23, 24). Single-center studies have major limitations, insufficient data heterogeneity, and many results may have varying degrees of overfitting (25). Multi-center research can provide diversified imaging data, which can better interpret the heterogeneity of tumors and conform to the development of precision medicine. The prediction model must be verified by independent external data to truly accurately evaluate its effectiveness. The advantage of this research lies in the collection of data from multiple hospitals. The largest data set in one hospital was used as the training cohort, and the data from the other two hospitals were merged into an independent validation cohort. Radiomics research needs to undergo repeated tests in multiple centers with large samples in order to accurately and reliably guide clinical medical strategies.

This study has a number of limitations: 1. Sample size is relatively small, and the multi-center data is limited to China. In the future, it is necessary to conduct international multi-center research; 2. We did not include the clinical characteristics of GISTs, but only constructed a pure radiomics prediction model. This is mainly due to the fact that in previous studies, the radiomics model has been shown to be superior to both the clinical index model and the subjective CT findings model. In fact, the three sets of parameters are correlated to different degrees, and radiomics can realize the deep mining and utilization of medical image data (8). 3. This is a retrospective study. The sample selection is biased and requires verification by a prospective study.

## Conclusion

Radiomics technology can effectively extract CT image representations of GISTs with different risk levels. The three-class GISTs risk stratification prediction model constructed based on it showed excellent predictive performance, and its generalization ability was also verified in external independent data. Radiomics has the potential to become a digital biopsy technique for preoperative assessment of the risk stratification of GISTs, helping clinicians to accurately stratify GISTs patients and identify the best treatment plan for precision treatment.

## Data Availability Statement

The datasets presented in this article are not readily available. Requests to access the datasets should be directed to chzhh149@sina.com.

## Ethics Statement

The patient consent to review their medical records was waived because our study was a retrospective non-interventive study, which did no harm to patients. Written informed consent for participation was not required for this study in accordance with the national legislation and the institutional requirements.

## Author Contributions

ZC, YX, LX, and HCC: conception and design, writing, review, and revision of the manuscript. MW, ZF, and CH: analysis and interpretation of data. ZC: study supervision. All authors contributed to the article and approved the submitted version.

## Conflict of Interest

CH was employed by the company Deepwise & League of PHD Technology Co., Ltd.

The remaining authors declare that the research was conducted in the absence of any commercial or financial relationships that could be construed as a potential conflict of interest.
